# Impact of war-associated factors on spread of sexually transmitted infections: a systemic review

**DOI:** 10.3389/fpubh.2024.1366600

**Published:** 2024-04-05

**Authors:** Yulia Kvasnevska, Mariia Faustova, Kseniia Voronova, Yaroslav Basarab, Yaroslava Lopatina

**Affiliations:** ^1^AIDS Healthcare Foundation, Kyiv, Ukraine; ^2^Microbiology, Virology and Immunology Department, Poltava State Medical University, Poltava, Ukraine

**Keywords:** sexually transmitted infections, war-associated factors, HIV, armed conflicts, war

## Abstract

**Introduction:**

Statistical data indicate a link between war and the spread of sexually transmitted infections (STIs), then it is necessary to carefully analyze the factors that directly affect the identified pattern in order to overcome this problem. Therefore, the purpose of the study was to systematically analyze the factors that influence the spread of STIs during war.

**Methods:**

The study included all original research articles and meta-analyses on the impact of war on the spread of sexually transmitted infections that met the following eligibility criteria: (1) articles published exclusively in English; (2) articles published in the period 2013–2023; (3) studies with quantitative, qualitative or mixed design. The search for relevant literature was conducted using four databases: PubMed, Embase, Web of Science, and Ebsco.

**Results:**

The articles selected for our systematic review had different research designs and were mainly published as original studies (*n* = 8) and literature reviews (*n* = 6). As a result of the evaluation of the selected articles for the systematic review, the authors identified migration, a decrease in access to health care, difficult access to contraception, sexual violence as the most frequent factors directly affecting the spread of STIs during the war.

**Conclusion:**

This systematic review systematizes data on the impact of hostilities on the spread of STIs and outlines the main factors that contribute to the dissemination of pathogens far beyond the territory at the epicenter of the conflict.

**Systematic review registration:**https://www.crd.york.ac.uk/prospero/display_record.php?ID=CRD42023479808, CRD42023479808.

## 1 Introduction

Full-scale wars and localized armed conflicts have a devastating impact on society and lead to critical material and human losses (1). According to the Uppsala Conflict Data Program (UCDP), as of 2021, just over 50 armed conflicts took place in the world during the twenty-first century. Moreover, it was believed that the period after the end of World War II was the most peaceful time in human history, during which there were no massive interstate wars (2). However, the morning of February 24, 2022, when military aircraft of another state took off over the capital of Ukraine, fundamentally refuted this statement (3). This was followed almost immediately by an escalation of the Israeli-Palestinian conflict. In this regard, society once again faced the challenges that arise from wars and the consequences they leave behind.

While the direct health consequences of armed conflict, such as injuries and mortality, are obvious and subject to constant monitoring and statistics, the indirect ones often remain hidden. Indirect health effects include post-traumatic stress, exacerbation and spread of infectious diseases (3, 4). Armed conflict is considered an important cause of the burden of the HIV and other sexually transmitted infections (STIs) epidemic (5).

It is known that STIs after World War I became the second most common cause of disability among U.S. Army personnel, second only to military injuries and concussions (6). Modern approaches to the prevention of sexually transmitted diseases and methods of armed confrontation have somewhat changed the structure of STI infection. The historically known syphilis and gonorrhea have been supplemented by a large number of other viral and bacterial agents, such as HIV, hepatitis B, chlamydia, human papillomavirus, and others (7). However, the tendency to increase the level of infection with sexually transmitted pathogens during conflicts continues to this day. For example, after the civil war in Guinea Bissau, HIV prevalence more than doubled, and in Uganda it increased by 14% (8, 9). Since the outbreak of the armed conflict between Russia and Ukraine in 2014, HIV incidence in some parts of Ukraine has increased by more than 15%, with statistics from some regions still unavailable due to the occupation (10). These developments and the humanitarian crisis caused by the war jeopardize plans to reduce the incidence of STIs and achieve the 90–90-90 goal of ending the HIV pandemic globally.

If statistical data indicate a link between war and the spread of STIs, then in order to overcome this problem, it is necessary to carefully analyze the factors that directly affect the identified pattern. Therefore, the purpose of the study was to systematically analyze the factors that influence the spread of STIs during conflicts.

## 2 Materials and methods

This systematic review was conducted in accordance with The Preferred Reporting Items for Systematic Reviews and Meta-Analyses (PRISMA) statement and approved by the International Register of Prospective Systematic Reviews (PROSPERO), CRD42023479808, November 17, 2023.

### 2.1 Eligibility criteria

The study included all original research articles and meta-analyses on the impact of war on the spread of sexually transmitted infections that met the following eligibility criteria: (1) articles published exclusively in English; (2) articles published in the period 2013–2023; (3) studies with quantitative, qualitative or mixed design. Exclusion criteria were: (1) conference abstracts, dissertations, posters; (2) articles published before 2013; (3) articles in languages other than English; (4) articles that did not correspond to the research topic; (5) articles whose full text version is not available.

### 2.2 Sources of information and search strategy

The search for relevant literature was conducted using four databases: PubMed, Embase, Web of Science, and Ebsco.

The search strategy used for the PubMed database is presented in Table 1. This search query was adapted and applied to all databases used in the study.

**Table 1:** Search strategy for PubMed.

No	Request
1	(armed conflicts[MeSH Terms] OR (armed[All Fields] AND conflicts[All Fields]) OR armed conflicts[All Fields] OR war[All Fields]) AND (sexually transmitted diseases[MeSH Terms] OR (sexually[All Fields] AND transmitted[All Fields] AND diseases[All Fields]) OR sexually transmitted diseases[All Fields])
2	(“sexually transmitted infection*” [Title/Abstract] OR “sexually transmitted disease*” [Title/Abstract]) AND (“war” [Title/Abstract] OR “arm*” OR “conflict” [Title/Abstract])

### 2.3 Data extraction

First, an initial search of all databases was conducted, followed by the removal of duplicates. Next, three authors (MF, YaB and YaL) independently screened articles by title and abstract. Articles that did not meet the eligibility criteria were excluded. At the end of this step, a collegial expert meeting was held to discuss the articles for which there were disagreements in the authors’ decisions regarding acceptance for further work or rejection.

At the next stage, one author (YuK) extracted the necessary elements from the selected articles into a pre-designed standardized data extraction form. The final version of the data was checked and approved by all members of the research team. The data extraction form included: (1) DOI or PMID of the article; (2) First and last names of the authors; (3) Title of the article; (4) Year of publication; (5) Country included in the study; (6) Study design; (7) Period of the study; (8) Main findings on the impact on STI spread.

All data obtained were double-checked 1 month after the initial extraction to optimize the reliability of the internal estimator and minimize the risk of bias.

The quality assessment was conducted using the Critical Appraisal Checklist for Studies Reporting Prevalence Data, which was developed and validated by the Joanna Briggs Institute (11). It consists of 9 questions, to which researchers can answer “yes,” “no,” “unclear” or “not applicable (NA)” for each item. The more “no” or “not sure” are selected, the greater the risk of bias in each category and in each study. The critical appraisal was conducted taking into account the variables of interest in our review. This step was also performed by two independent and previously trained researchers (MF and YaB), with a third researcher (YaL) always consulted in case of disagreement.

## 3 Results

### 3.1 Characteristics of the included studies

**Figure 1:**
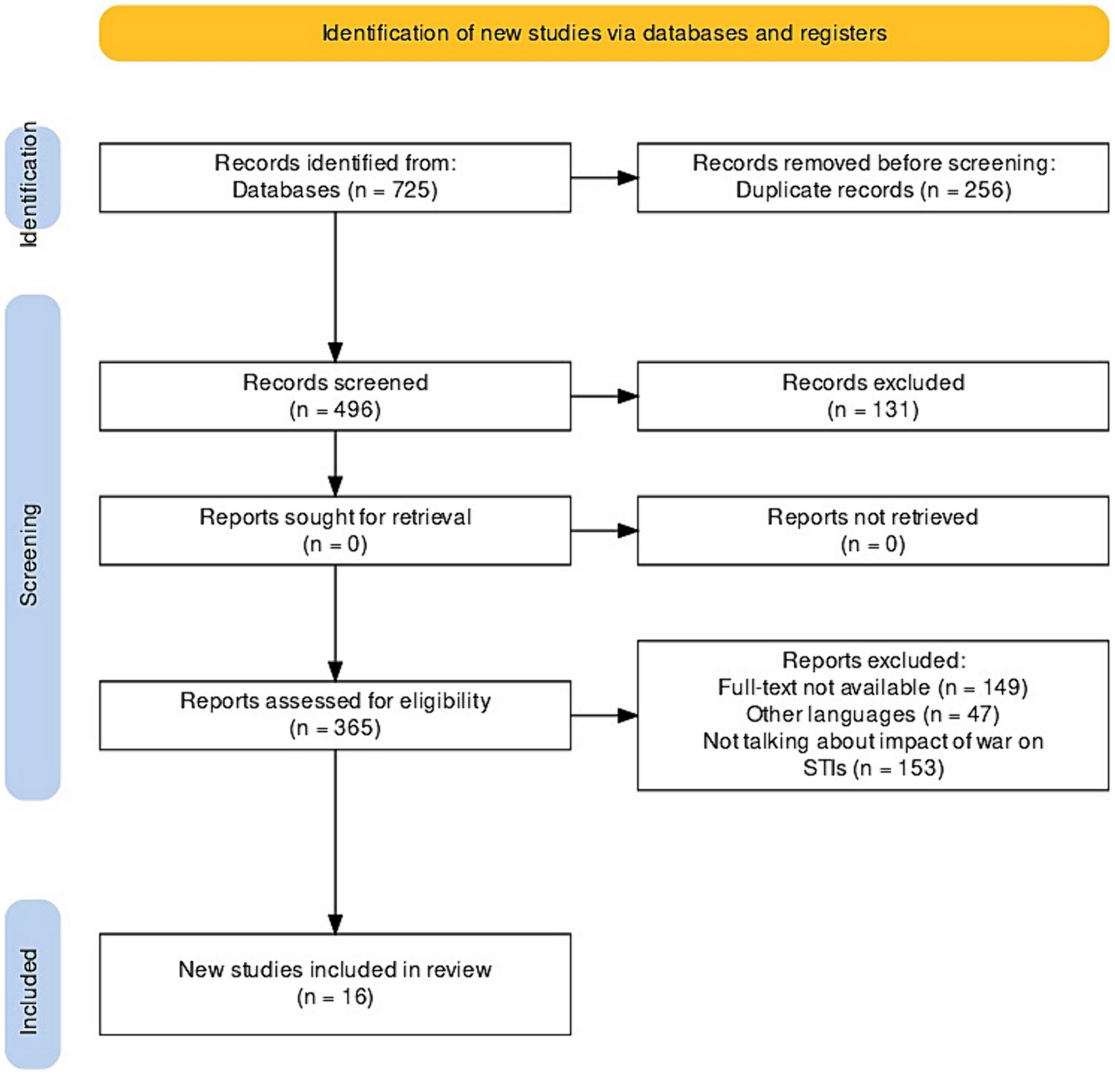
Flowchart of study selection according to the PRISMA guideline.

A total of 752 publications were identified through database searches. After removing duplicates (*n* = 256), the articles were evaluated by titles and abstracts for relevance to the research topic. The authors identified 365 articles, of which 149 were excluded due to the lack of full-text versions, 47 due to publication in languages other than English, and 153 due to the lack of information on the impact of war on the spread of STIs. At the end of the selection process, 16 articles were retained for full-text review and evaluation and subsequent inclusion in the systematic review. The study selection process is presented in Figure 1 (12).

The articles selected for our systematic review had different research designs and were mainly published as original studies (*n* = 8) and literature reviews (*n* = 6). We also included such publications as correspondence (*n* = 1) and perspectives (*n* = 1). The selected studies were published in 2016 (*n* = 3), 2021 (*n* = 3), 2022 (*n* = 3), 2023 (*n* = 3), 2018 (*n* = 2), 2015 (*n* = 1), and 2020 (*n* = 1). The largest number of articles were from the United States and Ukraine (3 from each country), 2 from Uganda, and one publication each from Austria, the United Kingdom, Greece, Ethiopia, the Netherlands, Rwanda, Libya, and Spain A general description of the selected articles and the main factors of the war’s impact on the spread of STIs described in them are presented in Supplementary Table S1.

### 3.2 Factors influencing the spread of STIs during war

**Figure 2:**
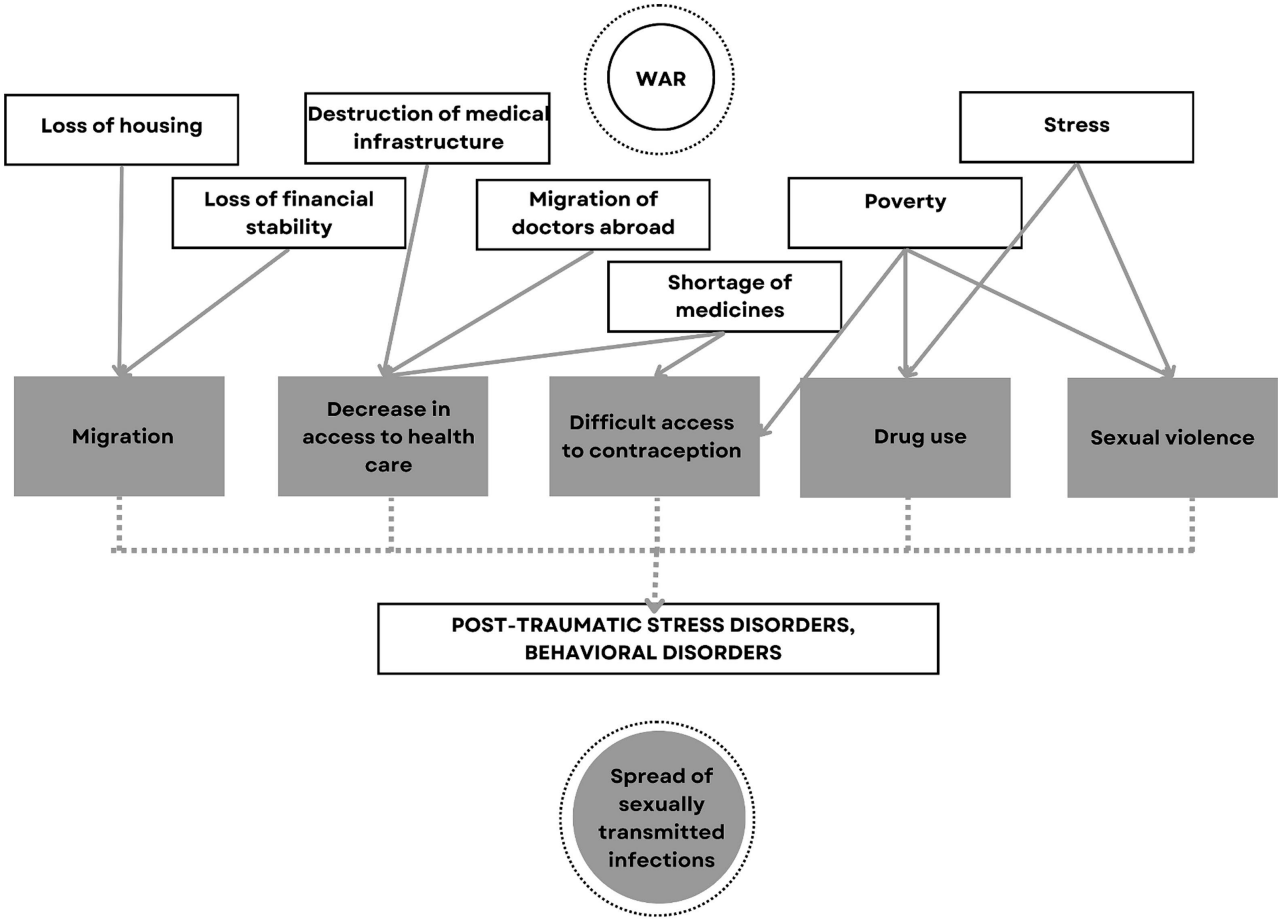
Flowchart of war-associated factors effecting the STIs.

As a result of the evaluation of the selected articles for the systematic review, the authors of 11 articles (5, 8, 10, 13–20) out of 16 included in the systematic review identified migration as the most frequent factor directly affecting the spread of STIs during the war (Figure 2).

Even from a historical perspective, data show an increase in the incidence and spread of STIs during World War II, which is primarily associated with uncontrolled sexual intercourse between military personnel in different regions of Europe and beyond (14, 16). For example, literature data indicate an increase in the dynamics of HIV spread in Libya during the Second Civil War. After all, when changing their place of residence, temporarily displaced persons can spread new viral strains (8). Moreover, some female internally displaced persons, having lost financial support, resort to sex work as the most affordable and only source of subsistence (18, 19). According to a survey of sex workers as representatives of key populations for HIV in Odesa, competition has increased significantly since the beginning of the war in Ukraine, especially given the fact that internally displaced persons often offer services at reduced prices, including without condoms (18). In general, poverty and the stress of changing place of residence significantly reduce the adherence of HIV-positive people to treatment or even contribute to its temporary discontinuation. First of all, this leads to an increase in viral load and higher infectiousness of patients. However, at the same time, disruption of treatment regimens with non-nucleoside reverse transcriptase inhibitors, which are currently used in Ukraine for most first-line regimens, can lead to mutations and the development of viral resistance within a few weeks (10, 20).

Particular attention should be paid to the external international migration of the population of countries in armed conflicts. For example, more than 5 million Ukrainians, mostly women and children, left the country during the first months after the outbreak of full-scale war in 2022. Given the fact that heterosexual contact in Ukraine has been identified as the dominant mode of HIV transmission, a significant number of those infected (more than 120,000) are women. And this is a matter of concern for the European Union countries, which have accepted the lion’s share of Ukrainian refugees (19). The long stay of women who have left their families and are left without means of subsistence facilitates the search for partners in the host country and, undoubtedly, the spread of infections. Moreover, the migration itself often lasts several days in overcrowded vehicles, during which refugees are exposed to extreme conditions of cold or heat, malnutrition, stress and poor hygiene, which cannot but affect their health and reactivation of persistent infections (13). That is why countries hosting refugees fleeing war must take swift and effective action to respond to STI screening, especially among key populations.

Another frequent factor associated with the war that affects the spread of STIs is a decrease in access to health care in 10 publications (10, 13–17, 19–22) out of 16. For example, research in Uganda in the post-war period showed that about 20% of HIV-positive women faced problems with access to health care related to their status (21). This is primarily due to the destruction of medical infrastructure in the country with active hostilities (13). According to official data, 120 medical institutions in Ukraine were damaged in the first week of Russia’s full-scale invasion alone, 10 of which were completely destroyed (3). And throughout the war in the Tigray region of Ethiopia, 70% of local hospitals were destroyed (17). At the same time, health care facilities that continue to operate in wartime face a significant number of challenges that affect the availability, speed, and quality of health care. For example, the reduction in the number of hospitals, the concentration of people in safer regions, and the migration of doctors abroad lead to overloading of health care facilities (20). At the same time, there is a high probability of a shortage of medicines, including ART, diagnostic tests, and medical supplies due to disrupted supply lines both within the country and from abroad. It is worth noting that doctors themselves point to the fact that much less attention is paid to the diagnosis, prevention and monitoring of STIs in wartime due to the overload of medical facilities with critical patients with war-related injuries (20).

Important factor that influenced the spread of STIs during the war was identified as difficult access to contraception in 9 articles (8, 10, 14, 15, 17, 18, 21, 23, 24) out of 16 selected for the systematic review. Despite the fact that armed conflicts and the stress associated with them lead to a decrease in sexual desire and, consequently, a decrease in sexual intercourse, meeting the needs of the population for contraception is an important key to preventing the spread of sexually transmitted diseases (18). As the experience of countries such as Libya, Ethiopia, Rwanda, Uganda, and Ukraine, which have experienced war in recent years, shows, a significant portion of the population does not have free access to condoms (15, 17, 18, 21). This is primarily due to the destruction of logistics chains and the occupation of territories, which makes it difficult or impossible to supply free contraceptives, and the decline in the population’s ability to pay makes it impossible to buy them, if they are available (18, 20).

For example, according to studies after the civil war in Libya, only 39% of young people surveyed indicated the importance and regularity of condom use, and 21% of MSM belonging to key populations used a condom during their last sexual intercourse (15). A similar study in the post-war period in Rwanda showed that only 6.1% of women who did not know their HIV status used condoms in the vast majority of sexual intercourse. Moreover, the same study found an inverse correlation between the HIV status of women who reported sexual activity in the past 6 months and the frequency of condom use (23). A similar pattern has been found in Ukraine since the beginning of the armed conflict among people who use drugs. The authors found that condom use during sexual intercourse with casual partners is associated with fewer cases of imported infections (19, 20).

Despite the fact that people who use drugs are among the key populations in peacetime, 7 articles (5, 8, 15, 20–22, 24) among those selected for the systematic review indicated an increase in HIV incidence in this category of patients during conflicts. For example, after the civil war in Libya, 87% of people who use drugs were HIV-positive, which was a world record at the time (15). It is worth noting that literature data pointed to the prevalence of injecting HIV transmission in Muslim countries where armed confrontations took place, as opposed to, for example, Ukraine, where the main mode of transmission is sexual (8). The direct correlation between the increase in STI incidence and injecting drug use can undoubtedly be linked to the increase in the number of such people amid the hostilities. After all, internal displacement, change of social circle, stress, and the desire to “hide from reality” contribute to the first attempts at drug use. The situation is significantly aggravated by the problem of access to disposable syringes during the occupation of the territories (10, 20). At the same time, drug use can have an indirect impact on the spread of STIs. For example, American researchers have established an interesting pattern that with the increase in the use of psychoactive substances (alcohol, drugs, etc.), the link between armed conflict and HIV increases. In other words, factors that arise in the context of conflict, such as stress, changes in living conditions and sexual behavior in the presence of drug and alcohol use, increase the susceptibility of the population to STIs, including HIV (5).

Particular attention should be paid to rape, the frequency of which increases significantly during armed conflicts. That is why this factor in the spread of STIs was mentioned in 6 (8, 9, 17, 21–23) of the 16 articles included in the systematic review. After the civil war in Rwanda in 1994, the term “genocidal rape” was coined, when sexual violence was used as a weapon against women of all ages for a long time. According to research in Rwanda, more than 50% of the 800 women who took part in the survey had been raped (9, 23). According to the literature, the population of all countries in which armed confrontation is unfolding is exposed to sexual violence. For example, in Ethiopia, the percentage of raped women reached 10%, in Sierra Leone - 8%, and in Ukraine - 2.6%. However, it is clear that these figures are much higher in reality, as a significant number of women and girls often do not confess to sexual torture (17). There is no doubt that rape, sometimes repeated or gang rape, is directly related to the possibility of STI transmission. However, there is also an indirect relationship between them. According to statistics, rape survivors often have post-traumatic stress disorders related to sexual behavior, which in turn can lead to unconscious risky sexual intercourse and infection or transmission to the following partners (23).

## 4 Discussion

The fact that war has an irreversible destructive effect on various aspects of human life is obvious. In addition to the direct impact on the life and health of the population, military actions have an effect on the political, socio-economic situation. This creates a so-called vicious circle, since the decrease in economic indicators and social protection of people again shifts the vectors of influence in the direction of public health (25, 26). This burden falls on the shoulders not only of military personnel who are directly in the epicenter of the war, but also on the civilian population. Thus, according to the literature, during the 20th century, the number of war-related deaths totaled more than 190 million people, and almost half of them were civilians (27, 28).

**Figure 3:**
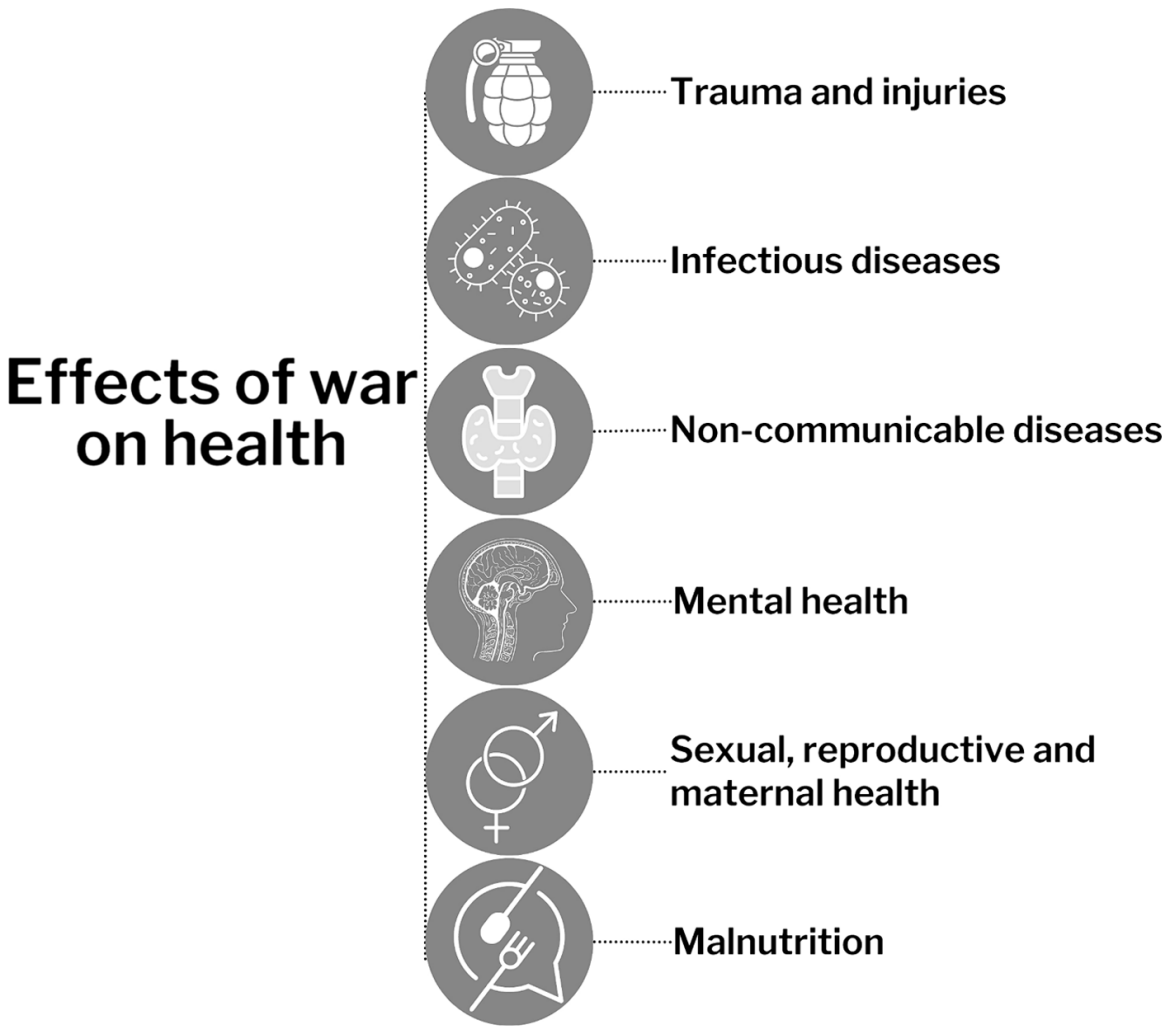
Flowchart of effects of war on health.

The direct effect of war on the morbidity and mortality of the population is associated with the use of firearms, biological, chemical and nuclear weapons, bombings, missile attacks, which lead to trauma and injuries (Figure 3) (28, 29). According to the data of past wars the death rate after the use of weapons varies depending on the tactics of fighting and the type of weapon used. After all, the experience of previous wars shows that bombing and missile attacks lead to an increase in the level of injuries and mortality, compared to one-on-one contact combat (29, 30). Recently, a significant part of military actions is precisely non-contact combat, for example, massive missile attacks and the use of unmanned aerial vehicles on the territory of Ukraine. This leads to the development of shrapnel, burn wounds and very often - mass death of people under rubble (25, 29). Along with this, war has a hidden effect on public health, worsening the situation with diseases not directly related to hostilities. Literature data indicate that the ratio of direct and indirect deaths during the war is 1:9. However, such conclusions do not have a strong evidence base. After all, recording the indirect effects of war on health is complex and often underestimated (28, 31). Moreover, some of the information may be classified or contain state secrets in the post-war period, and some aspects related to STI transmission may be downplayed or hidden due to religious prohibitions in certain countries (32).

The frequency of endemic and epidemic infectious diseases increases in wartime against the background of permanent stress, a decrease in the resistance of the body, malnutrition, deterioration of hygiene and a decrease in access to medical care (28, 33). Crowding of people in cramped spaces that are usually poorly ventilated (basements, shelters, etc.) contribute to the rapid spread of respiratory infections, while shortages of food and drinking water lead to the consumption of poor-quality products and, accordingly, outbreaks of intestinal disorders. Even vaccine-controllable infections can become active in times of war due to disruptions in drug supply chains and mass population migration. Thus, during the blockade of Syrian cities during the civil war, an outbreak of measles was observed in 2017–2018. Although previously, since 1999, this disease did not appear on the territory of the country (33). It is worth adding that in the context of infectious diseases, an important stumbling block is the rapid development of antibiotic resistance. After all, military injuries are very contaminated and often require urgent empirical use of antimicrobial agents on the battlefield. In addition, the lack of medicines and the medical supervision among civilians also further contributes to the uncontrolled use of antibiotics and, accordingly, the development of antimicrobial resistance (3, 34, 35).

The level of non-communicable diseases is globally high in peacetime and the increase in their frequency during wartime is underestimated compared to injuries and infectious diseases (28, 36). Chronic stress, malnutrition, excessive smoking, sleep and mental disorders, along with the lack of systemic adequate management of patients with chronic respiratory diseases, diabetes, cardiovascular diseases, and cancer, significantly increase the risk of severe complications or death. The experience of the wars in Bosnia and Syria showed an increase in death from non-communicable diseases both among women and men during and in the post-war period (37, 38).

A real “time bomb” can be considered mental disorders that are formed during the war, but can appear rather slowly for a long time even after the cessation of hostilities (25). Constant fear for one’s life and the lives of loved ones, loss of housing and financial stability, migration, observation of deaths and injuries, exposure to violence contribute to the development of stress, insomnia, behavioral disorders and post-traumatic stress disorders (25, 28, 39). A very often unnoticed factor in the development of mental disorders in children are family problems arising during the war. As a certain part of families break up due to long-term separation of parents, use of alcohol or drugs by one of the partners to “evade” reality, etc. Against the background of constant stress, a vulnerable child’s psyche experiences such situations excessively acutely, which can result in serious mental disorders in the future (25, 40).

This systematic review demonstrates the most important factors contributing to the spread of STIs during the war, because there is no doubt that their frequency is increased along with infectious diseases. The vast majority of publications addressed the impact of population migration, difficult access to health care and contraceptives, drug use, and sexual violence as factors that increase the transmission of STIs during hostilities. Along with this, we found only one study in Georgia, which showed no mathematical relationship between the increase in the frequency of STI diagnosis and population migration in the post-war period (41). Therefore, this fact requires further detailed research. Recent research in Liberia has been interesting and correlates with the data highlighted in the review regarding the increase in STIs in the context of war. Moreover, the authors indicate a significant dependence of the development of STIs s with socio-economic problems arising during war, exposure to trauma, sexual violence, stress and depression (42).

Undoubtedly, the implementation of classical STI prevention measures used in peacetime in military medicine should have yielded positive results. For example, the rate of STI infection among Polish special forces personnel who have been sexually active in recent months remained low due to their high awareness of protection methods and the full provision of condoms to their units (32). However, unfortunately, this is currently an exception rather than a systematic practice, as military prevention programs are extremely challenging (43, 44). Moreover, as the above factors show, the problem of STI spread during war concerns not only military personnel, but also the civilian population of the country, which can often pose a threat far beyond the borders of the state in which the conflict has unfolded (13, 45). That is why the issue of prevention and reduction of STI prevalence during conflicts is a global problem that all countries should be involved in solving. After all, national plans to combat STIs, including HIV, in the European Union are based on their own epidemiological experience and are aimed mainly at working with MSM and migrants from underdeveloped countries to a greater extent than with heterosexual women. Therefore, today it is worth shifting the focus to this new target group (19, 46). First and foremost, health care workers and community activists who are in direct contact with newly arrived refugees from war-torn countries should actively offer tests, preferably free of charge, and provide easy communication with health care facilities (19, 47).

Since the outbreak of full-scale war on the territory of Ukraine in 2022, neighboring countries that have hosted the vast majority of refugees have been continuously taking steps to actively involve displaced persons in the local health care system. Countries such as Poland, Romania, Moldova, Germany, Portugal, and others have enrolled Ukrainian IDPs in state health care programs, providing almost a full range of services, including access to free ART and HIV screening (20, 48). However, the statistics on STI-related medical care in these countries were extremely low (20). A rather serious problem in this context is the fear of stigmatization in a new team/society, disclosure of confidential information about HIV status, injecting drug use, or sexual activity. However, no less important is the temporary shift in values from caring for one’s health to the basic human needs of livelihood, housing, food, etc. The language barrier also plays a role, as many refugees literally do not seek help because they do not know the language (49).

Wars always lead to a humanitarian crisis, which, in turn, slows down both the country’s development and the pace of achieving national plans and strategies (50–52). Therefore, an important element of combating the spread of STIs in the world during war should be the rational and correct distribution of the burden of the epidemic of these diseases among all countries by reducing stigma, creating free access to medical care and testing. After all, the more people who know their status in a timely manner and receive qualified care, the higher the chances of achieving the goal of eliminating or reducing the level of STI infection.

## 5 Limitations

Given the constant updating of scientific data on STIs, a selection of data by topic for the last 10 years was carried out, which could potentially discard a certain part of the studies.

Moreover, the limitation was the inclusion of only English-language sources of literature and only those that were available in the databases in the full-test version.

In addition, there was a potential bias in the selection of articles for the literature review, despite the authors’ efforts to avoid this by independent review of the publications by three individuals.

A limitation may be the technique of quickly reviewing texts for compliance with the study design, which may have affected the depth and comprehensiveness of the review.

Prospectively, it is interesting to investigate this topic in the form of a meta-analysis in order to clarify the statistical data of the influence of certain factors on the spread of STIs, which was not covered within the scope of this study.

## 6 Conclusion

This systematic review summarizes and systematizes data on the impact of hostilities on the spread of STIs and outlines the main factors that directly or indirectly contribute to the dissemination of pathogens far beyond the territory at the epicenter of the conflict.

By assessing the challenges faced by countries involved in war, systemic solutions should be organized to potentially improve the situation:

- first of all, the unification of efforts of public and state organizations to establish an uninterrupted supply of medicines, contraceptives and rapid tests for the diagnosis of STIs in the territory of war.
- the deployment of state programs of psychological support for the population affected by the war, with the aim of timely detection of mental disorders and prevention of their complications.
- along with the supply of essential drugs (antibiotics, insulin, etc.), pay attention to the critical need for patients to receive antiretroviral therapy systemically, a break in the reception of which can lead to the development of resistance.
- to organize high-quality medical support for refugees in host countries with the aim of timely testing for STIs and providing support to patients.
- identification of persons, especially children and adolescents, who were subjected to sexual violence, followed by their psychological support.
- create barrier-free access to medical care.

## Data availability statement

The raw data supporting the conclusions of this article will be made available by the authors, without undue reservation.

## Author contributions

YK: Data curation, Formal analysis, Methodology, Writing – review & editing. MF: Formal analysis, Methodology, Software, Visualization, Writing – original draft, Writing – review & editing. KV: Data curation, Formal analysis, Writing – original draft. YB: Conceptualization, Data curation, Project administration, Writing – review & editing. YL: Conceptualization, Data curation, Methodology, Project administration, Supervision, Validation, Writing – original draft, Writing – review & editing.

## References

[1] Khorram-Manesh A Burkle FM Goniewicz K Robinson Y. Estimating the number of civilian casualties in modern armed conflicts-a systematic review. Front Public Health. (2021) 9:765261. doi: 10.3389/fpubh.2021.765261, PMID: 34778192 PMC8581199

[2] Bendavid E Boerma T Akseer N Langer A Malembaka EB Okiro EA . The effects of armed conflict on the health of women and children. Lancet. (2021) 397:522–32. doi: 10.1016/S0140-6736(21)00131-8, PMID: 33503456 PMC7612212

[3] Loban' G Faustova M Dobrovolska O Tkachenko P. War in Ukraine: incursion of antimicrobial resistance. Ir J Med Sci. (2023) 192:2905–7. doi: 10.1007/s11845-023-03401-x, PMID: 37178279 PMC10182337

[4] Sundaram M Filion A Akaribo BE Stephens PR. Footprint of war: integrating armed conflicts in disease ecology. Trends Parasitol. (2023) 39:238–41. doi: 10.1016/j.pt.2023.01.007, PMID: 36803860 PMC10194412

[5] Kerridge BT Saha TD Hasin DS. Armed conflict, substance use and HIV: a global analysis. AIDS Behav. (2016) 20:473–83. doi: 10.1007/s10461-015-1161-4, PMID: 26286341 PMC4760915

[6] Wilson SE Roudiez C DeSomer H Lewis C Yetter N. Vicious habits: sexually transmitted infections among Black and white union Army veterans. J Appl Hist. (2020) 1:53–67. doi: 10.1163/25895893-00101003, PMID: 33123681 PMC7592704

[7] Gottwald C Schwarz NG Frickmann H. Sexually transmitted infections in soldiers - a cross-sectional assessment in German paratroopers and navy soldiers and a literature review. Eur J Microbiol Immunol. (2019) 9:138–43. doi: 10.1556/1886.2019.00023, PMID: 31934366 PMC6945994

[8] Daw MA El-Bouzedi AH Ahmed MO. The impact of armed conflict on the prevalence and transmission dynamics of HIV infection in Libya. Front Public Health. (2022) 10:779778. doi: 10.3389/fpubh.2022.779778, PMID: 35433583 PMC9009867

[9] Spittal PM Malamba SS Ogwang MD Musisi S Ekwaru JP Sewankambo NK . Cango Lyec (healing the elephant): gender differences in HIV infection in post-conflict northern Uganda. J Acquir Immune Defic Syndr. (2018) 78:257–68. doi: 10.1097/QAI.0000000000001671, PMID: 29509587 PMC6012052

[10] Vasylyeva TI Liulchuk M Friedman SR Sazonova I Faria NR Katzourakis A . Molecular epidemiology reveals the role of war in the spread of HIV in Ukraine. Proc Natl Acad Sci USA. (2018) 115:1051–6. doi: 10.1073/pnas.1701447115, PMID: 29339468 PMC5798316

[11] Munn Z Moola S Riitano D Lisy K. The development of a critical appraisal tool for use in systematic reviews addressing questions of prevalence. Int J Health Policy Manag. (2014) 3:123–8. doi: 10.15171/ijhpm.2014.71 25197676 PMC4154549

[12] Haddaway NR Page MJ Pritchard CC McGuinness LA. PRISMA2020: an R package and shiny app for producing PRISMA 2020-compliant flow diagrams, with interactivity for optimised digital transparency and open synthesis. Campbell Syst Rev. (2022) 18:e1230. doi: 10.1002/cl2.1230, PMID: 36911350 PMC8958186

[13] Padovese V Knapp A. Challenges of managing skin Diseases in refugees and migrants. Dermatol Clin. (2021) 39:101–15. doi: 10.1016/j.det.2020.08.010, PMID: 33228854

[14] Stary A. The changing Spectrum of sexually transmitted infections in Europe. Acta Derm Venereol. (2020) 100:adv00114. doi: 10.2340/00015555-3470, PMID: 32207537 PMC9128896

[15] Hamidi A Regmi PR van Teijlingen E. HIV epidemic in Libya: identifying gaps. J Int Assoc Provid AIDS Care. (2021) 20:23259582211053964. doi: 10.1177/23259582211053964, PMID: 34841956 PMC8640281

[16] Tsiamis C Vrioni G Poulakou-Rebelakou E Gennimata V Murdjeva MА Tsakris A. Medical and social aspects of syphilis in the Balkans from the mid-19th century to the interwar. Folia Med. (2016) 58:5–11. doi: 10.1515/folmed-2016-0001, PMID: 27383872

[17] Fisseha G Gebrehiwot TG Gebremichael MW Wahdey S Meles GG Gezae KE . War-related sexual and gender-based violence in Tigray, northern Ethiopia: a community-based study. BMJ Glob Health. (2023) 8:e010270. doi: 10.1136/bmjgh-2022-010270, PMID: 37479499 PMC10364179

[18] Friedman SR Smyrnov P Vasylyeva TI. Will the Russian war in Ukraine unleash larger epidemics of HIV, TB and associated conditions and diseases in Ukraine? Harm Reduct J. (2023) 20:119. doi: 10.1186/s12954-023-00855-1, PMID: 37658448 PMC10472698

[19] Jonas KJ Parczewski M Davidc v d V. The war refugees from Ukraine: an HIV epidemic is fleeing as well. AIDS. (2022) 36:1745–6. doi: 10.1097/QAD.0000000000003271 36052542

[20] Vasylyev M Skrzat-Klapaczyńska A Bernardino JI Săndulescu O Gilles C Libois A . Unified European support framework to sustain the HIV cascade of care for people living with HIV including in displaced populations of war-struck Ukraine. Lancet HIV. (2022) 9:e438–48. doi: 10.1016/S2352-3018(22)00125-4, PMID: 35576942

[21] Muyinda H Jongbloed K Zamar DS Malamba SS Ogwang MD Katamba A . Cango Lyec (healing the elephant): HIV prevalence and vulnerabilities among adolescent girls and young women in Postconflict northern Uganda. J Acquir Immune Defic Syndr. (2023) 94:95–106. doi: 10.1097/QAI.0000000000003234, PMID: 37276188 PMC10497204

[22] Alzate Angel JC Pericàs JM Taylor HA Benach J. Systemic factors and barriers that hamper adequate data collection on the HIV epidemic and its associated inequalities in countries with long-term armed conflicts: lessons from Colombia. Am J Public Health. (2018) 108:1341–4. doi: 10.2105/AJPH.2018.304505, PMID: 30138065 PMC6137797

[23] Adedimeji AA Hoover DR Shi Q Gard T Mutimura E Sinayobye J . Sexual behavior and risk practices of HIV positive and HIV negative Rwandan women. AIDS Behav. (2015) 19:1366–78. doi: 10.1007/s10461-014-0964-z, PMID: 25488169 PMC4461563

[24] Callands TA Gilliam SM Sileo KM Taylor EN Hunter-Jones JJ Hansen NB. Examining the influence of trauma exposure on HIV sexual risk between men and women in post-conflict Liberia. AIDS Behav. (2021) 25:1159–70. doi: 10.1007/s10461-020-03088-6, PMID: 33180254 PMC7979480

[25] Goniewicz K Burkle FM Dzhus M Khorram-Manesh A. Ukraine’s healthcare crisis: sustainable strategies for navigating conflict and rebuilding for a resilient future. Sustain For. (2023) 15:11602. doi: 10.3390/su151511602

[26] Khorram-Manesh A Burkle FM Jr. Civilian population victimization: a systematic review comparing humanitarian and health outcomes in conventional and hybrid warfare. Disaster Med Public Health Prep. (2022) 17:e192. doi: 10.1017/dmp.2022.96 35400358

[27] Heisler M Iacopino V. War is a global threat to public health. BMJ. (2019) 365:l4031. doi: 10.1136/bmj.l4031, PMID: 31171543

[28] Garry S Checchi F. Armed conflict and public health: into the 21st century. J Public Health. (2020) 42:e287–98. doi: 10.1093/pubmed/fdz095 31822891

[29] Coupland RM Meddings DR. Mortality associated with use of weapons in armed conflicts, wartime atrocities, and civilian mass shootings: literature review. BMJ. (1999) 319:407–10. doi: 10.1136/bmj.319.7207.407, PMID: 10445920 PMC28193

[30] Gates S. Hegre H. Mokleiv Nygård H. Strand H. (2010). Consequences of civil conflict (2010). World Development Report 2011 Background Paper. Washington, DC: The World Bank.

[31] Roberts A. Lives and statistics: are 90% of war victims civilians? Survival. (2010) 52:115–36. doi: 10.1080/00396338.2010.494880

[32] Kim Y. Armed conflict, health spending, and HIV. Int J Health Plann Manag. (2018) 33:581–95. doi: 10.1002/hpm.2499 29468739

[33] The Lancet Infectious Diseases. War and infectious diseases: brothers in arms. Lancet Infect Dis. (2022) 22:563. doi: 10.1016/S1473-3099(22)00235-3, PMID: 35405091

[34] Ljungquist O Nazarchuk O Kahlmeter G Andrews V Koithan T Wasserstrom L . Highly multidrug-resistant gram-negative bacterial infections in war victims in Ukraine, 2022. Lancet Infect Dis. (2023) 23:784–6. doi: 10.1016/S1473-3099(23)00291-8, PMID: 37236220

[35] Nazarchuk O Faustova M Kolodii S. Microbiological characteristics of infectious complications, actual aspects of their prevention and treatment in surgical patients. Novosti Khirurgii. (2019) 27:318–27. doi: 10.18484/2305-0047.2019.3.318

[36] The Lancet. Non-communicable diseases: what now? Lancet. (2022) 399:1201. doi: 10.1016/S0140-6736(22)00567-0, PMID: 35339211 PMC9755319

[37] Poole D. Indirect health consequences of war: cardiovascular disease. Int J Sociol. (2012) 42:90–107. doi: 10.2753/IJS0020-7659420205

[38] Ramadan H Naja F Fouad F Antoun E Jaffa M Chaaban R . Prevalence and correlates of metabolic syndrome in pre-crisis Syria: call for current relief efforts. East Mediterr Health J. (2016) 22:668–75. doi: 10.26719/2016.22.9.668, PMID: 27966768

[39] Bogic M Njoku A Priebe S. Long-term mental health of war-refugees: a systematic literature review. BMC Int Health Hum Rights. (2015) 15:1–41. doi: 10.1186/s12914-015-0064-9 26510473 PMC4624599

[40] Osokina O Silwal S Bohdanova T Hodes M Sourander A Skokauskas N. Impact of the Russian invasion on the mental health of adolescents in Ukraine. J Am Acad Child Adolesc Psychiatry. (2022) 62:335–43. doi: 10.1016/j.jaac.2022.07.845 36441074

[41] Doliashvili K Buckley CJ. Women's sexual and reproductive health in post-socialist Georgia: does internal displacement matter? Int Fam Plan Perspect. (2008) 34:021–9. doi: 10.1363/ifpp.34.021.08, PMID: 18440914

[42] Callands TA Taylor EN Sileo KM Gilliam SM Hansen NB. Understanding the effects of trauma exposure, life stress, intimate partner violence, and depression on sexually transmitted infection risk in post-conflict Liberia. Arch Sex Behav. (2024) 53:1519–30. doi: 10.1007/s10508-023-02765-6, PMID: , 38167991

[43] Korzeniewski K. Urogenital *Chlamydia trachomatis* in the environment of soldiers from the polish special forces. Ann Agric Environ Med. (2019) 26:51–4. doi: 10.26444/aaem/85591, PMID: 30922029

[44] Duron S Bohet A Panjo H Bajos N Migliani R Marimoutou C . Sexual health in the French military: a multidimensional and gendered perspective. BMC Public Health. (2018) 18:750. doi: 10.1186/s12889-018-5571-x, PMID: 29914422 PMC6007003

[45] Anastario MP Hallum-Montes R Reyes E Manzanero R Chun H. Toward a social theory of sexual risk behavior among men in the armed services: understanding the military occupational habitus. Cult Med Psychiatry. (2013) 37:737–55. doi: 10.1007/s11013-013-9335-x, PMID: 24101537

[46] Murray CJL Lopez AD Tomijima N. Armed conflict as a public health problem. BMJ. (2002) 324:346–9. doi: 10.1136/bmj.324.7333.346 11834565 PMC1122272

[47] ECDC. HIV/AIDS surveillance in Europe 2021 (2020 data). Available at: https://www.ecdc.europa.eu/en/publications-data/hiv-aids-surveillance-europe-2021-2020-data. (Accessed December 4, 2023).

[48] Lichtenheld AG. Explaining population displacement strategies in civil wars: a cross-national analysis. Int Organ. (2020) 74:253–94. doi: 10.1017/S0020818320000089

[49] Wolters Kluwer Polish government ACT of 12 march 2022 on assistance to Ukrainian citizens in connection with an armed conflict in the territory of that country. https://sip.lex.pl/akty-prawne/dzu-dziennik-ustaw/pomoc-obywatelom-ukrainy-w-zwiazku-z-konfliktem-zbrojnym-na-terytorium-19216115?_ga=2.175776738.1767019042.1648243897-1252350147.1648243897 date (Accessed December 4, 2023).

[50] Broqua C Laborde-Balen G Menetrier A Bangoura D. Queer necropolitics of asylum: Senegalese refugees facing HIV in Mauritania. Glob Public Health. (2021) 16:746–62. doi: 10.1080/17441692.2020.1851744, PMID: 33275869

[51] UNAIDS Office on AIDS and Humanitarian Response. HIV/AIDS and conflict. Copenhagen: UNAIDS Office on AIDS and Humanitarian Response (2003).

[52] WHO. WHO response to the Ukraine crisis. Geneva: WHO (2022). 2022 p.

