# Supplementary material for: Impact of war-associated factors on spread of sexually transmitted infections: a systemic review
Source: Front Public Health. 2024 Apr 5;12:1366600. doi: 10.3389/fpubh.2024.1366600 (PMC11026856; doi:10.3389/fpubh.2024.1366600)
Supplement: Supplementary file 1 [file Table_1.DOCX]

Supplementary Material

# Supplementary Table S1

**Characteristics of the studies included in the systemic review**

| Authors | Year | Country | Article type | Access to care | Conditions during travel and the living conditions | Malnutrition | Screening policies | Torture | Chronic, noninfectious diseases | Poverty | Access to contraception | Migration | Reduction of prostitution prices | Young age | Rape | Drug use | War trauma |
| --- | --- | --- | --- | --- | --- | --- | --- | --- | --- | --- | --- | --- | --- | --- | --- | --- | --- |
| Padovese V, Knapp A. | 2021 | USA | Review | + | + | + | + | + | + |  |  | + |  |  |  |  |  |
| Stary A. | 2020 | Austria | Review | + |  |  | + |  |  | + | + | + |  |  |  |  |  |
| Hamidi A, Regmi PR, van Teijlingen E. | 2021 | UK | Review | + |  |  |  |  |  |  | + | + | + |  |  | + |  |
| Tsiamis C, Vrioni G, Poulakou-Rebelakou E, *et al.* | 2016 | Greece | Review | + |  |  |  |  |  |  |  | + | + |  |  |  |  |
| Friedman, S.R., Smyrnov, P. & Vasylyeva, T.I. | 2023 | Ukraine | Perspective |  |  | + |  |  |  | + | + | + | + | + |  |  |  |
| Fisseha G, Gebrehiwot TG, Gebremichael MW, *et al.* | 2023 | Ethiopia | Original research | + |  |  |  | + |  |  | + | + |  | + | + |  |  |
| Jonas KJ, Parczewski M, van de Vijver D. | 2022 | Netherands | Correspond. | + |  |  | + |  |  |  |  | + |  |  |  |  |  |
| Vasylyev M, Skrzat-Klapaczyńska A, Bernardino JI, *et al.* | 2022 | Ukraine | Review | + | + |  | + |  |  |  |  | + |  |  |  | + |  |
| Adedimeji AA, Hoover DR, Shi Q, *et al.* | 2015 | Rwanda | Original research |  |  |  |  | + |  |  | + |  |  | + | + |  |  |
| Callands TA, Gilliam SM, Sileo KM, *et al.* | 2021 | USA | Original research |  |  |  |  | + | + |  | + |  |  | + |  | + |  |
| Vasylyeva TI, Liulchuk M, Friedman SR, *et al.* | 2018 | Ukraine | Original research | + |  |  |  |  |  |  | + | + |  |  |  |  |  |
| Daw MA, El-Bouzedi AH, Ahmed MO. | 2022 | Libya | Original research |  |  |  |  |  |  |  | + | + | + |  | + | + |  |
| Kerridge BT, Saha TD, Hasin DS. | 2016 | USA | Original research |  | + |  |  |  | + |  |  | + |  |  |  | + | + |
| Spittal PM, Malamba SS, Ogwang MD. *et al.* | 2018 | Uganda | Original research |  |  |  |  |  | + | + | + |  |  |  | + |  |  |
| Muyinda H, Jongbloed K, Zamar DS, *et al.* | 2023 | Uganda | Original research | + |  |  |  |  | + |  |  |  |  |  | + | + | + |
| Alzate Angel JC, Pericàs JM, Taylor HA, Benach J. | 2018 | Spain | Review | + |  |  | + |  |  |  |  |  |  |  | + |  |  |
